# Scrotal hematoma: a rare complication of transfemoral percutaneous coronary intervention

**DOI:** 10.1186/s43044-024-00552-9

**Published:** 2024-09-06

**Authors:** Jaafar S. Aldoori, Araz Abdulfaraj, Shahla M. S. Rasul

**Affiliations:** 1 Department of Cardiology, Slemani Cardiac Hospital (SCH), Qanat Street, Sulaymaniyah, Kurdistan Region 46001 Iraq; 2 Department of Radiology, College of Medicine, Sulaymaniyah University, Sulaymaniyah, Kurdistan Region Iraq

**Keywords:** Transfemoral approach, Percutaneous coronary intervention, Scrotal hematoma, Case report

## Abstract

**Background:**

Cardiac catheterization via the transfemoral approach can be associated with access site bleeding complications such as inguinal hematoma, pseudoaneurysm, arteriovenous fistula and retroperitoneal hematoma. Scrotal hematoma is a rare presentation of bleeding complications after transfemoral cardiac catheterization. We report a case of this rare complication.

**Case presentation:**

A 63-year-old male with previous coronary artery bypass surgery underwent percutaneous coronary intervention via transfemoral approach. Few hours after removal of the femoral sheath, he developed a big scrotal hematoma and hemodynamic deterioration. The patient responded successfully to conservative treatment and discharged from hospital after three days in a stable condition.

**Conclusions:**

Bleeding complications after transfemoral cardiac catheterization can rarely present as scrotal hematoma. The management of this complication is usually conservative, and only few cases may require surgical treatment.

## Background

The transfemoral approach (TFA) for cardiac catheterization is well known for its bleeding complications such as inguinal hematoma, pseudoaneurysm, arteriovenous fistula (A-V) and retroperitoneal hematoma. Although inguinal hematoma is a common manifestation of access site bleeding after TFA, scrotal or penoscrotal hematoma is rare. Only few cases of scrotal hematoma were reported in the literatures [1–3]. We report a case of this rare complication.

## Case presentation

A 63-year-old male, hypertensive, non-diabetic, non-smoker, had 3-vessel coronary artery disease (CAD) with multiple percutaneous coronary interventions (PCI) and then coronary artery bypass graft (CABG) surgery in 2011. He presented with exertional chest pain and admitted for elective coronary angiography (CAG) with possible PCI.

Because the patient had a previous CABG surgery, we chose the TFA to perform the procedure. Diagnostic CAG was done via right femoral artery using six French (6F) femoral sheath followed by ad hoc PCI for left main stem (LMS)/ramus intermedius branch with implantation of two drug eluting stents (DES). The procedure was uneventful with good angiographic results. The patient received dual antiplatelet therapy (aspirin plus clopidogrel) but glycoprotein IIb/IIIa inhibitors were not used.

The femoral sheath removed 3 h after the procedure and hemostasis done by manual compression for 15 min. Subsequently, a sand bag was applied for 30 min over the groin according to our center protocol. After 4 h of bed rest, the patient was advised to mobilize when he suddenly collapsed and developed severe suprapubic and genital pain.

**Fig. 1:**
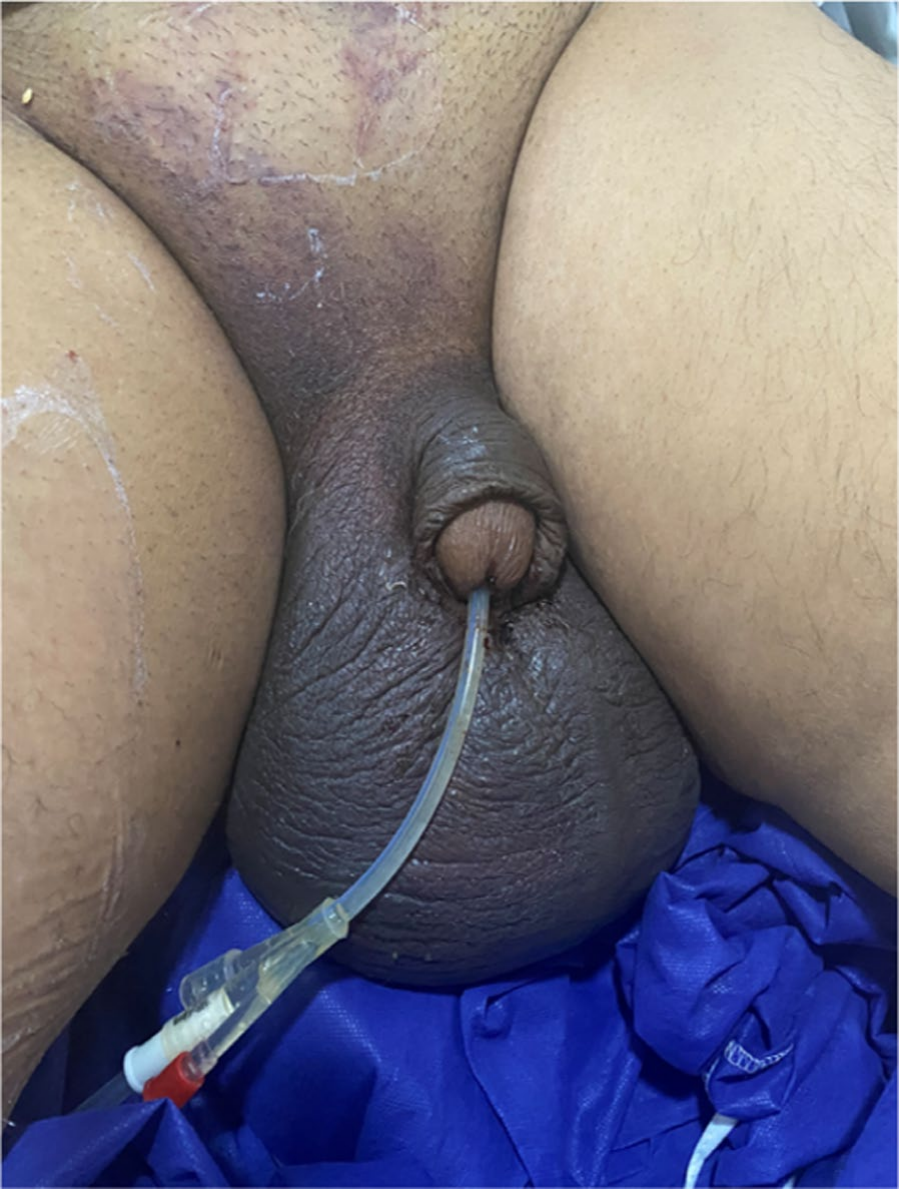
Big scrotal hematoma with no significant inguinal hematoma

On physical examination, the patient was pale, irritable, had profuse sweating, with tachycardia and blood pressure of 90/60 mmHg. There was a big scrotal swelling (Fig. 1) without visible femoral or inguinal hematoma or ooze from the puncture site.

His hemoglobin dropped from 12.4 to 10.2 g/dl. After rapid intravenous (IV) infusion of one pint of normal saline, the blood pressure raised to 110/70 mmHg. Because of significant drop in hemoglobin, the patient received one unit of packed red blood cells.

**Fig. 2:**
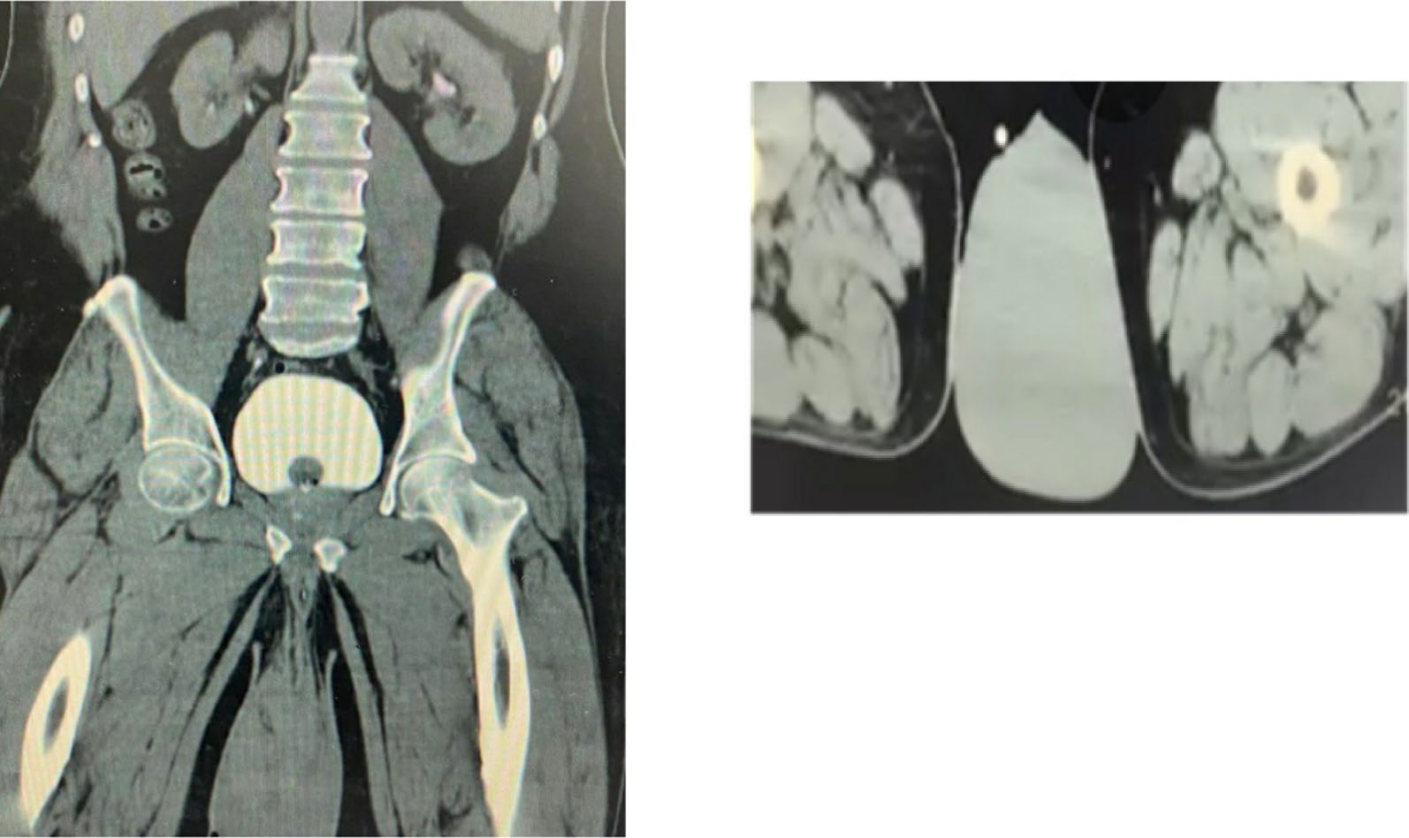
CT scan of the abdomen and pelvis; big scrotal hematoma around the right testis with stranding of the fat tissue and thickening of the soft tissue planes at the right inguinal region. No retroperitoneal hematoma

Computed tomography (CT) scan of the abdomen and pelvis revealed a big scrotal hematoma around the right testis with stranding of the fat tissue and thickening of the soft tissue planes at the right inguinal region. There was no retroperitoneal hematoma (Fig. 2).

Vascular surgery and urology consultation were done and both suggested conservative management with analgesia and bed rest.

**Fig. 3:**
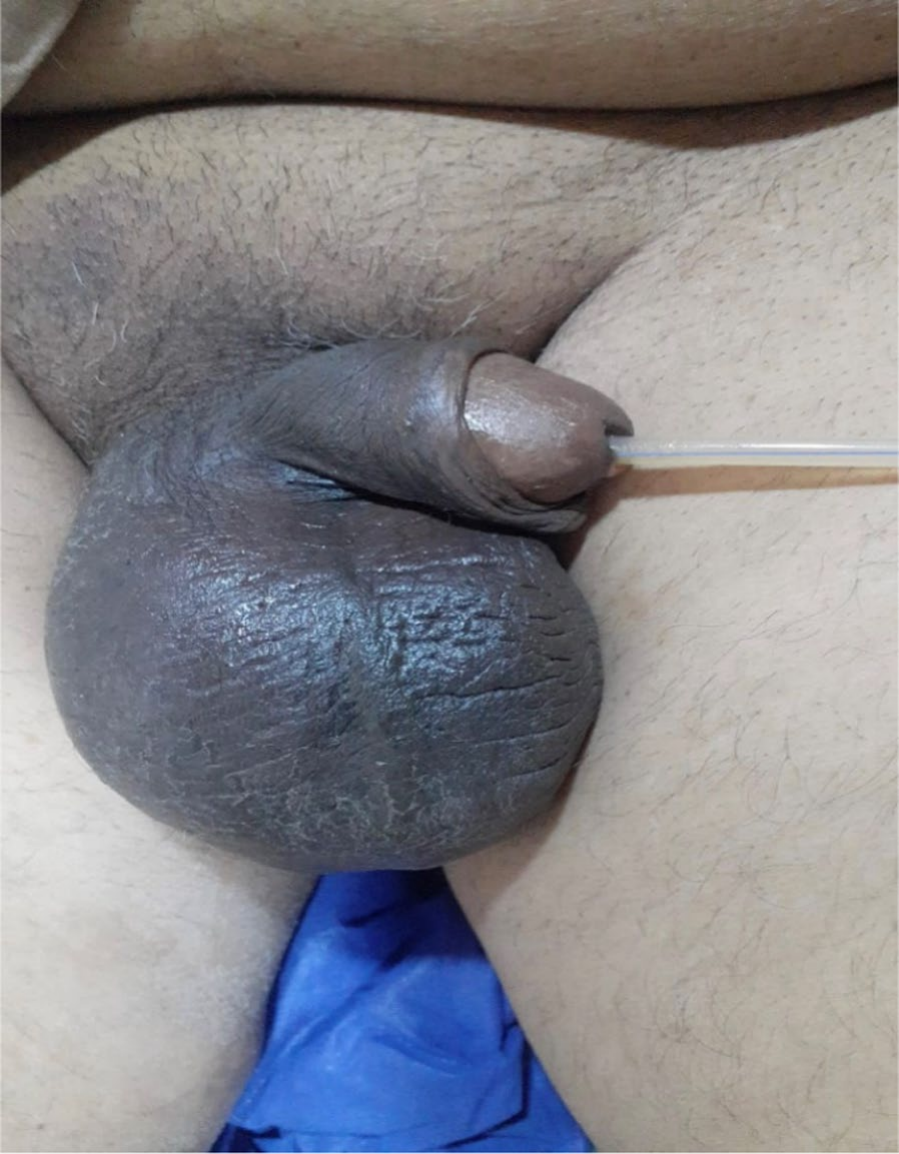
Reduction in the size of the scrotal hematoma after 2 days of conservative treatment

The patient responded well to conservative management and scrotal swelling decreased gradually in size (Fig. 3). He was discharged after 3 days to be seen after a week in the outpatient clinic.

**Fig. 4:**
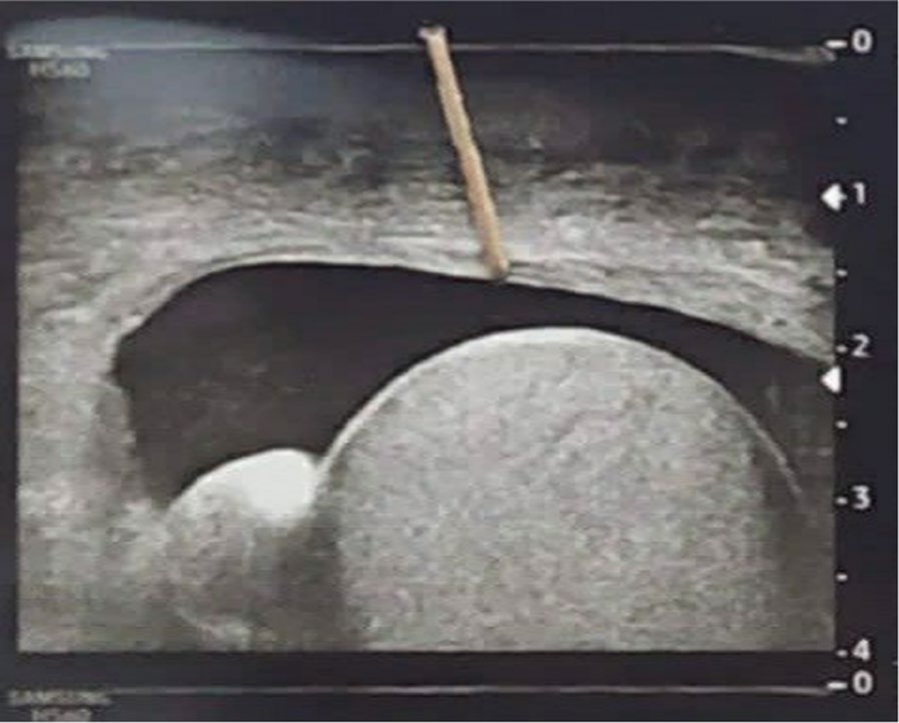
Ultrasound of the testis; small right-sided hematoma. Normal size and vascularity of right testis

At follow-up, ultrasound examination of the scrotum showed small right-sided hematoma, with normal size and vascularity of the right testis (Fig. 4).

## Discussion

The transradial approach (TRA) had globally replaced the TFA as the primary access for CAG and PCI since decades. However, the TFA may be preferred in certain clinical situations such as in patient with previous CABG surgery or patient with failed TRA. Bleeding is the most common complication of the TFA and can manifest as hematoma, uncontrolled bleeding, pseudoaneurysm or retroperitoneal hemorrhage [4]. The incidence of major bleeding complications after PCI ranges from 2 to 6% [4, 5]. While inguinal hematoma is common, scrotal hematoma is very rare and only few cases reported in the studies. Few cases of scrotal/penoscrotal hematoma were reported with failed vascular closure device (VCD) [1, 2]. A scrotal hematoma occurs when the femoral puncture is at or very close to the inguinal ligament, with blood tracking along the spermatic cord into the scrotum [3]. Bleeding above the inguinal ligament such as retroperitoneal hemorrhage and bleeding from the inferior epigastric vessel through the pre-peritoneal space both may dissect down into the spermatic cord and the inguinal canal, causing groin and scrotal hematoma [6–8].

The risk of access site bleeding is higher in female sex, older age, obese patient, renal impairment, interventional procedures as compared to diagnostic procedures, use of anticoagulation, increased sheath size and use of glycoprotein IIb/IIIa antagonists.

To reduce access site bleeding complications, several approaches can be adopted. In our case, the left TRA may be a good option to avoid the TFA with additional benefit, which is easier engagement of the left internal mammary artery in comparison with TFA.

When choosing TFA, puncturing the femoral artery at the proper site will greatly reduce access site complications. This should be at the mid common femoral artery (CFA) above its bifurcation into the deep femoral artery and the superficial femoral artery. Using the inguinal ligament as a landmark, the femoral artery should be punctured at the area of maximal pulsation, 2–3 cm below the mid inguinal ligament. At this course, the femoral artery lies in front of the middle third of the head of the femur and can be easily compressed against the bone to achieve hemostasis after sheath removal. High femoral puncture above the inguinal ligament is associated with higher risk of retroperitoneal hemorrhage, while very low puncture increases the risk of pseudoaneurysm or arteriovenous (A-V) fistula.

Fluoroscopic or ultrasound-guided femoral access might help in localizing the proper site for femoral artery cannulation and reduce bleeding complications. Fluoroscopic-guided TFA uses the femoral head as a reference with the aim to do the skin prick over the inferior border of the middle third of femoral head. Ultrasound-guided femoral puncture can precisely localize the CFA and therefore avoid high or low femoral access. A recent randomized clinical trial (The UNIVERSAL Trial) “Routine Ultrasonography Guidance for Femoral Vascular Access for Cardiac Procedures” compared ultrasonography-guided approach on top of fluoroscopy versus fluoroscopy without ultrasonography for TFA. This trial showed that routine use of ultrasonography did not reduce the primary events of bleeding or vascular complications, but ultrasonography did reduce the risk of venipuncture and number of attempts [9].

Various types of vascular closure devices (VCD) had been used to achieve hemostasis and to replace manual or mechanical compression, e.g., AngioSeal (St. Jude Medical), Prostar XL (Abbott Vascular), Perclose ProGlide (Abbott Vascular). These devices allowed early ambulation of the patients and faster discharge, but did not reduce access site bleeding complications [1, 3, 10].

After finishing the procedure, performing angiography through the sidearm of the arterial sheath will identify any dissection, perforation or retroperitoneal hematoma and allowing the operator to intervene immediately.

The management of scrotal hematoma is usually conservative with painkillers, IV fluid, blood transfusion (when the blood loss is significant leading to drop in patient’s hemoglobin or hemodynamic instability), bed rest, elevation of the scrotum and observation. Surgery is only indicated when there is active bleeding due to vascular injury, enlarging hematoma or the blood supply to the testes is compromised [1].

## Conclusion

Access site bleeding complications after transfemoral cardiac catheterization can rarely present as scrotal hematoma without associated inguinal hematoma. The scrotal hematoma can reach a big size and cause hemodynamic instability. The management of this complication is usually conservative, and only few cases may require surgical treatment.

## Data Availability

The datasets used and/or analyzed in this “case report” are available from the corresponding author on reasonable request.

## Abbreviations

A-V: Arteriovenous
CABG: Coronary artery bypass graft
CAD: Coronary artery disease
CAG: Coronary angiography
CFA: Common femoral artery
CT: Computed tomography
DES: Drug eluting stent
LMS: Left main stem
PCI: Percutaneous coronary intervention
TFA: Transfemoral approach
TRA: Transradial approach
VCD: Vascular closure device
